# Patient mistreatment of health care professionals

**DOI:** 10.1186/s12909-022-03198-w

**Published:** 2022-03-01

**Authors:** David A. Mahoney, Divya Gopisetty, Lars Osterberg, Matthew J. R. Nudelman, Rebecca Smith-Coggins

**Affiliations:** 1 Pediatrics Resident, Postgraduate Year 3, Stanford University School of Medicine, Stanford, CA USA; 2 Education Programs and Services, Stanford University School of Medicine, Stanford, CA USA; 3 Primary Care, Population Health, Stanford University School of Medicine, Stanford, CA USA; 4 Department of Pediatrics, Marshall University School of Medicine, Huntington, WV USA; 5 Department of Emergency Medicine, Stanford University School of Medicine, Stanford, CA USA; 6 Office of Medical Student Wellness, Stanford University School of Medicine, Stanford, CA USA

**Keywords:** Mistreatment, Disrespect, Curriculum, Victim, Bystander

## Abstract

**Background:**

Mistreatment of health care professionals by patients is an ongoing problem. We aimed to construct and evaluate a curriculum that would prepare health care professionals for mistreatment by patients.

**Methods:**

Lessons learned from 15 interviews and 2 focus groups with health care professionals were distilled into a multi-modal curriculum including didactics, simulation videos and role-play scenarios aimed to improve confidence in addressing mistreatment. This curriculum was disseminated at five educational workshops to health care professionals of various training groups and experience levels. Pre- and post-surveys were distributed to assess changes in participant’s perspectives on readiness to address mistreatment. The signed-rank test was implemented to compare pre- and post- data.

**Results:**

Participants were more likely to agree post-workshop that they had the right words to say, had a plan for what to do, and were more willing to speak up when they themselves or someone else was mistreated (*p* < .001). They were also more likely to agree post-workshop that there was something they could do to address patient mistreatment (*p* < .001).

**Conclusions:**

Participant familiarity and confidence in responding to patient mistreatment increased. Our curriculum may serve as a foundation for institutions seeking to equip their educators, health care professionals, and trainees with strategies for addressing this important issue.

**Supplementary Information:**

The online version contains supplementary material available at 10.1186/s12909-022-03198-w.

## Background

Health care professionals enter training with a deep desire to care for patients well. Unfortunately, patients are becoming an increasingly recognized source of mistreatment directed towards professionals, which is ultimately a threat to both patient and professionals’ well-being [1]. Mistreatment in the medical setting has broadly been defined as behavior that disrespects or disregards the dignity of another person [2]. Trainees are an especially vulnerable population given their place in the medical hierarchy, and recent literature would suggest addressing this issue is an urgent need [3, 4].

We now have extensive evidence that medical students suffer mistreatment throughout their training from a variety of sources [5] —in fact, there’s little question that health care professionals across all training levels are exposed to such abuse. One recent meta-analysis quantifying harassment and discrimination against both medical students and residents found that 59.4% of trainees experienced at least one form of abuse throughout their training, most commonly verbal [6]. Experiencing such behavior has been shown to correlate with burnout, which is associated with suicidal ideation and thoughts of dropping out of medical school [7–9]. Attending physicians are no more immune than their junior colleagues, with one study stating 1 in 3 experience rudeness, dismissiveness, and aggressive behavior multiple times a week. Seven percent state that this behavior has led to medical mistakes [10].

One study out of the University of Alberta stated that of the 45% of respondents who had experienced intimidation, harassment, or discrimination during their family medicine residency training, 35% identified patients as the source of this mistreatment [11]. Several publications have offered strategies for how to address patient mistreatment of health care professionals effectively, with suggestions including depersonalization, exploring the patient’s perspective, and “calling it out" [2, 12, 13]. To our knowledge, there are no studies to date that assess the effectiveness of training materials aimed at helping health care professionals respond to patient mistreatment. The authors aimed to address this issue by first constructing such a curriculum, then, by disseminating it to a range of health care professionals through workshops, and finally, by quantitatively measuring differences in attendee confidence before and after participating through surveys. By surveying health care professionals across several different levels of training, we hoped to assess the relative impact of such a curriculum (as well as mistreatment itself) on professionals with varying levels of experience. More broadly, we hoped that by recognizing and actively discussing these instances of mistreatment we would reinforce the importance of a culture of mutual respect within the medical system.

## Methods

### Study and curriculum design

IRB approval was obtained through Stanford University’s Research Compliance Office for all components of this study prior to initiation. The project consisted of two phases. The purpose of the first phase was to elicit the experiences of Stanford Medicine health care professionals with mistreatment. This served both as a needs assessment and a means of describing previously utilized strategies for addressing mistreatment in our community. In this phase the authors completed a series of semi-structured interviews regarding patient mistreatment of medical staff (Appendix A: Semi-structured Interview Questions) with medical students, residents, attendings, nurses and other staff at Stanford Medical School, Stanford University Medical Center and Stanford Children’s Health. The interview template was written and reviewed by the primary research authors [DM, LO, RS-C,], and a single author [DM] conducted each interview. Participants were identified through a mixture of volunteer and purposive sampling—some individuals responded to paper advertisements posted throughout campus, while others were sought out for their expertise in this topic. Advertisements requested participation of individuals who had either personally experienced patient mistreatment or had witnessed it. The authors garnered descriptions of instances of mistreatment, elicited what strategies were used to address mistreatment (if any), and inquired if those strategies were viewed as effective or not by the participant. Fifteen individual interviews and two focus groups with 4 participants each were completed between November 2017 and May 2018. Interviews were recorded with participant permission. Verbatim transcription of these interviews and focus groups were completed using an online service (Rev.com) and reviewed for accuracy by a single author [DM]. Transcripts were de-identified and stored on an encrypted, password-protected computer.

The authors then moved to the second phase of the study. The purpose of the second phase was to construct an educational curriculum for health care professionals to more effectively address patient mistreatment based on these interviews. They also aimed to evaluate how effectively this curriculum increased participant confidence in addressing mistreatment. Poignant narratives describing incidents of mistreatment were used as the basis for four video scenarios and one paper case to be used in the curriculum. Each video demonstrated one or more outcomes based on differing strategies utilized to address mistreatment, which mirrored strategies that were described during the interview phase of the study. The video scenarios depicted instances of racism, sexism, homophobia, and violent/threatening behavior. Scripts, discussion questions, and learning objectives were written collaboratively by the primary authors, with details sufficiently changed to maintain interview participant and patient anonymity. Learning objectives were based upon strategies that were uniformly described as helpful during the interview phase of the study. Actors for the video scenarios were health care professionals from the Stanford Medical School community—a subset of these actors were financially compensated. Camera work, sound work, and video editing was completed by Stanford Video. The final curriculum consisted of a combination of background education on the topic via literature review, screening of video scenarios, and audience participation via group discussion as well as role-play exercises using the paper case scenario (videos available at this link: https://vimeo.com/channels/1659548). Mistreatment was broadly defined for participants as behavior that disregards or disrespects the dignity of another person. Physical violence was considered to be an extreme form of mistreatment and we shared de-escalation strategies in our curriculum and directed participants to individual hospital policies regarding violent behavior for further guidance [14].

### Participants and sampling

The authors distributed this curriculum to a wide range of health care professionals including medical students, residents, attendings, nurses, and other staff members between September 2018 and March 2019 via a series of educational workshops. There were five workshops in total spanning pre-clinical medical student didactic sessions, international conferences, and residency program didactic sessions. Workshops at the pre-clinical didactic sessions and residency program didactic sessions were completed following the request of the associated faculty supervisor or residency program director. The international conference workshop setting was applied to and accepted. Workshops lasted between 60 and 90 minutes.

Subjects were included in the analysis if they initiated both the pre- and post-test survey regardless of completing each question. A total of 172 subjects completed either the pre- or post-test survey. Twenty-five subjects were excluded due to lack of either pre- or post-test data. Four subjects were excluded because they had identical subject-identifier numbers which prevented us from accurately pairing their pre- and post-test. A total of 143 subjects completed both the pre- and post-test and were used for this analysis. Of the 143 subjects, 62% were medical students, 12% were residents, 20% were attendings, and 6% were in another category (including nurse, nurse practitioner, psychologist, and case manager).

### Data collection

Pre- and post-test surveys (both identical) were distributed to participants who agreed to participate in the research study. Ten Likert scale survey questions were iteratively rewritten until we reached author consensus that the questions reflected curriculum learning objectives (See Appendix B for Pre- and post-test survey). These learning objectives included increasing participant knowledge about patient mistreatment and institutional resources (Questions 10, 11, 15), increasing participant comfort in addressing mistreatment (Questions 6, 8, 9, 12, 13, 14), and participant generation of an action plan for future instances of mistreatment (Question 7). For the Likert scale, 1 meant “strongly disagree” and 5 meant “strongly agree.” Pre- and post-test surveys were paired using the participant’s date of birth (day/month) and the first letter of the city of their birth. The only other identifying information obtained from participants was their training level. Additional identifying information was not gathered to preserve participant anonymity.

### Analysis

Descriptive statistics were used to describe responses to questions. Signed-Rank Test was used to compared pre- and post-test responses, both for the pooled cohort and also after stratifying participants by training group.

## Results

**Fig. 1:**
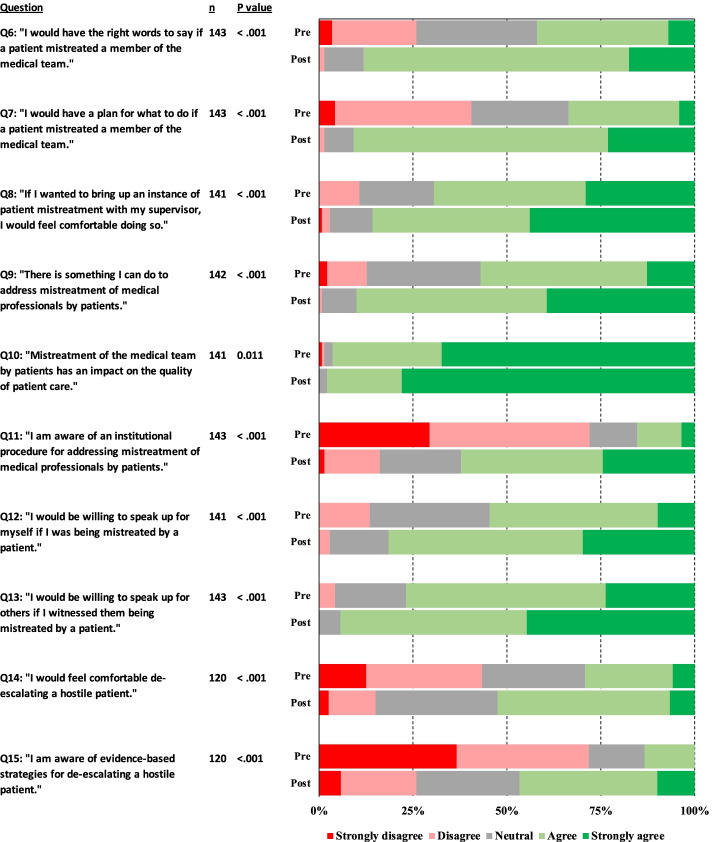
Pre- and Post-test response comparison by question, and *P*-value assessing for any change from pre- to post-test answers. Data is pooled from all participants

Pooled participants had a statistically significant increase (*p* < 0.001 for all questions) in post-response compared to their pre-test response for all questions six to fifteen (see Table 1 and Fig. 1). Question 16 asked whether participants preferred having an opportunity to address patient mistreatment themselves versus having their supervisor address the offending patient immediately. The percentage of respondents who preferred having an opportunity to address mistreatment themselves prior to supervisor intervention increased 18% following our educational intervention, from 31 to 49%. Notable post-test responses in the stratified analysis that did not differ significantly (*p* < 0.05) from the corresponding pre-test response included: comfort bringing up instances of mistreatment with supervisors amongst attendings (Q8), willingness to speak up for one’s self during an instance of mistreatment amongst residents (Q12), comfort and knowledge about utilizing evidence-based strategies to de-escalate a hostile patient amongst residents (Q14,15), and impression that mistreatment has an impact on the quality of patient care amongst all non-pooled groups (Q10). Participants in the “other” category, which included several non-patient-facing workers, rarely had post-test responses that differed significantly from pre-test responses.

## Discussion

Post-workshop, the pooled cohort of participants was more likely to agree that they had the right words to say, they had a plan for what to do, and they were willing to speak up when faced with mistreatment by patients (Fig. 1). Pooled participants also were more likely after the educational intervention to feel comfortable de-escalating a hostile patient and be aware of evidence-based de-escalation strategies. In addition, they were more likely to desire the opportunity to address mistreatment themselves rather than request immediate supervisor intervention following our educational intervention. These results suggest our curriculum bolstered the confidence of health care professionals across a range of training levels to find their voice in addressing patient mistreatment.

Regarding our stratified analysis, there are several possible explanations for lack of pre- and post-test responses differing significantly amongst various groups. Regarding question 10 (the only question in which medical students did not have a statistically significant difference in pre- and post-test response), lack of significance across all groups is most likely explained by the ceiling effect, as all groups already strongly agreed that mistreatment impacted quality of patient care. Regarding question 8, it’s possible that attendings don’t have an identified supervisor that is appropriate for discussing instances of mistreatment with compared to residents or medical students. Regarding question 12, lack of significant increase in resident willingness to speak up for oneself may be explained by the relatively low number of resident participants, and we are reassured by the trend of increased agreement. Similarly, lack of increase of resident comfort and knowledge surrounding hostile patient management was likely secondary to the very small number of residents that participated in workshops addressing this issue, with only 6 resident participants amongst 120 total respondents.

**Fig. 2:**
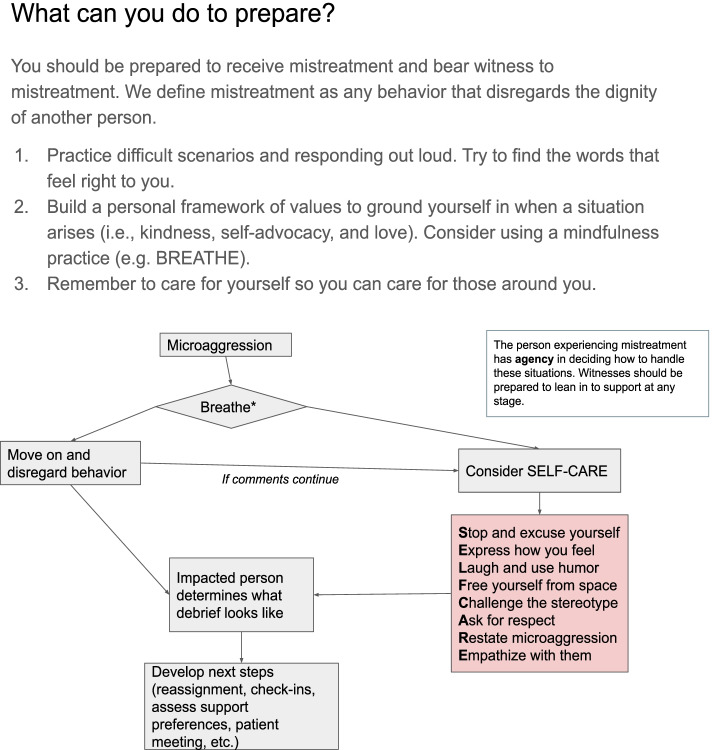
Proposed frameworks for preparing for and addressing patient mistreatment of medical staff

Our results suggest that mixed curricula like ours, which utilizes video depictions of simulated mistreatment, role-play scenarios, and development of planned responses to prejudice and inappropriate behavior, may be an effective option for medical institutions seeking to prepare health care professionals for the unfortunate inevitability of facing mistreatment. In our time holding these workshops, we frequently heard participants describe feeling frozen or paralyzed during instances of mistreatment, which oftentimes led to a sense of shame or regret for not being able to say something in the moment. Debriefing these scenarios may help participants find the words that feel right to them—such preparation may help overcome the initial shock of inappropriate behavior and “call out” mistreatment in a more timely (and potentially more satisfactory) manner [14]. This could be true both for the targets of mistreatment and for bystanders hoping to protect their peers or learners. Ultimately, we recommend the individual being mistreated be given agency to determine what the response to an instance of mistreatment looks like—this boosts the victim’s autonomy in addressing the event, and permits an educator or peer to tailor their support in a way that is most effective for the person being mistreated. Figure 2 summarizes strategies that interview and focus group participants deemed important to utilize when a medical team member experiences mistreatment. Educators may wish to incorporate other tools aimed towards addressing microaggressions, such as the GRIT mnemonic, or mindfulness tools such as BREATHE into their practice [15, 16].

The following limitations to our study should be noted. Questions 14 to 17 were on the back of a two-sided survey sheet and were left blank by 23 of our post-survey respondents. We suspect they were left blank because they were not seen by participants. We would not expect the respondents who did not see these final questions to have differed substantially from those who completed the survey, decreasing the likelihood this would bias our analysis. The post-intervention responses were obtained right after the educational intervention, and therefore it is unknown if these results would be sustained with time. It is possible that the participants over time would become less comfortable or would feel less prepared to address patient mistreatment. Repeating the post-test surveys at a later date to determine if the effects of the educational intervention are longer lasting would be a valuable assessment. Additionally, many of our participants were pre-clinical medical students with limited opportunity to have previously experienced patient mistreatment of health care professionals—future work would benefit from the involvement of students with more extensive clinical experience.

## Conclusions

Using trigger videos with patient mistreatment scenarios and role-playing these with medical trainees and faculty can prepare both trainees and faculty to more confidently handle these events when they occur. This has implications for improving patient care, given the ripple effect patient mistreatment of professionals has on quality of care. Further assessment of the longevity of increased confidence following curricular intervention, assessment of actual capability to address mistreatment following intervention, assessment of alternative tools, and assessment of the detrimental impact of mistreatment on health care professional wellbeing is warranted. A quantitative assessment of the prevalence of patient mistreatment of health care professionals should also be pursued. Of utmost importance, institutions should have robust infrastructure and processes in place to not only address patient mistreatment, but support their trainees, educators and staff who experience this type of abuse. Building a culture of mutual respect is critical for the well-being of both patients and the professionals caring for them. It is particularly important to have a heightened awareness for the potential for mistreatment of marginalized individuals given this population is at higher risk of being mistreated both within and beyond the medical setting. Future research in this area should include a dedicated exploration of the experience of marginalized health care professionals and their assessment of the effectiveness of the above strategies in addressing mistreatment.

**Table 1:** Pre and post test responses by participant group

					**Pre- and post-test difference**			
			**Pre-test**	**Post-test**				
**Question**	**Group**	**N**	**median (IQR)**	**median (IQR)**	**Decreased (n)**	**No change (n)**	**Increased (n)**	***P* value**
**Q7: "I would have a plan for what to do if a patient mistreated a member of the medical team."**	**Entire Cohort**	**143**	**3 (2, 4)**	**4 (4, 4)**	**5**	**50**	**88**	** < .001**
	**Medical Student**	87	3 (2, 4)	4 (4, 4)	4	20	63	< .001
	**Resident**	17	4 (3, 4)	4 (4, 4)	0	8	9	.003
	**Attending**	29	4 (3, 4)	4 (4, 5)	1	16	12	.002
	**Other**	10	4 (3, 4)	4 (4, 4)	0	6	4	.047
**Q6: "I would have the right words to say if a patient mistreated a member of the medical team."**	**Entire Cohort**	**143**	**3 (2, 4)**	**4 (4, 4)**	**1**	**32**	**110**	** < .001**
	**Medical Student**	87	2 (2, 3)	4 (4, 4)	1	13	73	< .001
	**Resident**	17	4 (3, 4)	4 (4, 5)	0	5	12	.001
	**Attending**	29	4 (3, 4)	4 (4, 5)	0	11	18	< .001
	**Other**	10	3 (3, 4)	4 (4, 4)	0	3	7	.008
**Q8: "If I wanted to bring up an instance of patient mistreatment with my supervisor, I would feel comfortable doing so."**	**Entire Cohort**	**141**	**4 (3, 5)**	**4 (4, 5)**	**14**	**71**	**56**	** < .001**
	**Medical Student**	87	4 (3, 4)	4 (4, 5)	8	37	42	< .001
	**Resident**	17	4 (4, 5)	5 (4, 5)	0	12	5	.025
	**Attending**	27	4 (4, 5)	4 (4, 5)	4	16	7	.376
	**Other**	10	4.5 (3, 5)	4 (4, 5)	2	6	2	.907
**Q9: "There is something I can do to address mistreatment of medical professionals by patients."**	**Entire Cohort**	**142**	**4 (3, 4)**	**4 (4, 5)**	**5**	**55**	**82**	** < .001**
	**Medical Student**	87	4 (3, 4)	4 (4, 5)	4	30	53	< .001
	**Resident**	17	4 (3, 4)	4 (4, 5)	0	8	9	.003
	**Attending**	29	4 (3, 4)	4 (4, 5)	0	14	15	< .001
	**Other**	9	3 (2, 5)	4 (3, 4)	1	3	5	.095
**Q10: "Mistreatment of the medical team by patients has an impact on the quality of patient care."**	**Entire Cohort**	**141**	**5 (4, 5)**	**5 (5, 5)**	**10**	**106**	**25**	**.011**
	**Medical Student**	86	5 (4, 5)	5 (5, 5)	6	66	14	.066
	**Resident**	17	5 (4, 5)	5 (5, 5)	0	14	3	.083
	**Attending**	29	5 (4, 5)	5 (4, 5)	4	20	5	.790
	**Other**	9	5 (4, 5)	5 (5, 5)	0	6	3	.083
**Q11: "I am aware of an institutional procedure for addressing mistreatment of medical professionals by patients."**	**Entire Cohort**	**143**	**2 (1, 3)**	**4 (3, 4)**	**5**	**33**	**105**	** < .001**
	**Medical Student**	87	2 (1, 2)	4 (3, 5)	1	10	76	< .001
	**Resident**	17	2 (2, 3)	4 (3, 4)	0	7	10	.002
	**Attending**	29	3 (2, 4)	4 (3, 5)	3	13	13	.012
	**Other**	10	2 (1, 3)	3 (3, 3)	1	3	6	.051
**Q12: "I would be willing to speak up for myself if I was being mistreated by a patient."**	**Entire Cohort**	**141**	**4 (3, 4)**	**4 (4, 5)**	**8**	**63**	**70**	** < .001**
	**Medical Student**	86	3 (3, 4)	4 (3, 4)	6	33	47	< .001
	**Resident**	17	4 (4, 4)	4 (4, 5)	1	10	6	.059
	**Attending**	29	4 (4, 4)	5 (4, 5)	1	17	11	.004
	**Other**	9	3 (3, 4)	4 (4, 4)	0	3	6	.014
**Q13: "I would be willing to speak up for others if I witnessed them being mistreated by a patient."**	**Entire Cohort**	**143**	**4 (4, 4)**	**4 (4, 5)**	**4**	**82**	**57**	** < .001**
	**Medical Student**	87	4 (3, 4)	4 (4, 5)	3	48	36	< .001
	**Resident**	17	4 (4, 5)	5 (4, 5)	0	10	7	.008
	**Attending**	29	4 (4, 5)	5 (4, 5)	1	18	10	.007
	**Other**	10	4 (4, 5)	4.5 (4, 5)	0	6	4	.046
**Q14: "I would feel comfortable de-escalating a hostile patient."**	**Entire Cohort**	**120**	**3 (2, 4)**	**4 (3, 4)**	**8**	**48**	**64**	** < .001**
	**Medical Student**	81	2 (2, 3)	3 (3, 4)	3	28	50	< .001
	**Resident**	6	4 (3, 4)	4 (4, 4)	1	3	2	.493
	**Attending**	25	4 (3, 4)	4 (4, 4)	3	11	11	.030
	**Other**	8	4 (3, 4)	4 (3, 4)	1	6	1	1.000
**Q15: "I am aware of evidence-based strategies for de-escalating a hostile patient."**	**Entire Cohort**	**120**	**2 (1, 3)**	**3 (2, 4)**	**3**	**28**	**89**	** < .001**
	**Medical Student**	81	1 (1, 2)	3 (2, 4)	2	16	63	< .001
	**Resident**	6	3 (3, 4)	4 (4, 5)	0	3	3	.086
	**Attending**	25	3 (2, 3)	4 (4, 4)	0	7	18	< .001
	**Other**	8	3 (2, 4)	4 (3, 4.5)	1	2	5	.094

*IQR* Interquartile Range (25^th^, 75^th^ percentile), *Q* Question number

Legend: 1 = strongly disagree, 2 = disagree, 3 = neutral, 4 = agree, 5 = strongly agree

## Supplementary Information

**Additional file 1.**

**Additional file 2.**

## Data Availability

The datasets used and/or analyzed during the current study are available from the corresponding author on reasonable request.
