# Supplementary material for: Patient mistreatment of health care professionals
Source: BMC Med Educ. 2022 Mar 1;22:133. doi: 10.1186/s12909-022-03198-w (PMC8886904; doi:10.1186/s12909-022-03198-w)
Supplement: Supplementary file 1 — Additional file 1. [file 12909_2022_3198_MOESM1_ESM.pdf]

Appendix: Semi-structured Interview Questions

PART 1—BASICS, EXISTING POLICIES

- How have you defined patient mistreatment of medicals staff previously, in your other project? Why did you chose to define it that way?
- What policies about patient behavior exist that you are aware of?
- What are the options you are aware of to report mistreatment by a patient?
- What options of reporting or policies can you envision, or do you think would be desirable?
- Who should broach the patient's mistreatment with the patient?
- How does the medical illness of the patient play into the interaction?
- What responsibilities do staff members have to each other in addressing mistreatment of medical staff, if any?

PART 2—SPECIFIC TO YOUR FIELD/AREA OF INTEREST

- Could you talk a little bit about your previous research in this area?
- How would you describe the current culture surrounding patient mistreatment of medical staff at LPCH?
- Is there any training currently for residents on how to deal with patient mistreatment? What would that look like, if there was one?
- How does mistreatment impact the wellness of staff?
- What strategies would you offer to medical staff, particularly residents or other trainees in terms of how they should deal with patient mistreatment, including both in the moment and after the fact?
    - How do those strategies differ depending on the training level of the individual being mistreated, if at all?
- How does mistreatment in a pediatric setting differ from that in an adult setting, if at all?

PART 3—PERSONAL INSTANCES OF MISTREATMENT

- Have you ever experienced mistreatment by a patient or witnessed mistreatment? Tell me about that experience.
- What part of that situation do you believe was handled well or led to a more positive outcome?
- What part of that situation was not handled well or led to a more negative outcome? How could that have been improved?
- How did mistreatment impact your personal well-being or the well-being of those around you?
- If you have never experienced or witnessed mistreatment, what do you think would be some strategies the person being mistreated could use to handle the situation? What about bystanders?
- If you have never experienced or witnessed mistreatment, what would be some strategies that should be avoided?
