# Supplementary material for: Patient mistreatment of health care professionals
Source: BMC Med Educ. 2022 Mar 1;22:133. doi: 10.1186/s12909-022-03198-w (PMC8886904; doi:10.1186/s12909-022-03198-w)
Supplement: Supplementary file 2 — Additional file 2. [file 12909_2022_3198_MOESM2_ESM.pdf]

Q1 Date of Workshop
Q2 Location of Workshop

Mistreatment Workshop Questionnaire
Q3 Birthdate (DD/MM) ____
Q4 First letter of the city you were born in (A) ____
Q5 Training Level/Discipline (Medical Student, Resident, Attending, Nurse, etc.): __________

Rate your level of agreement to the following statements on a scale of 1 to 5, with 1 being "strongly disagree" and 5 being "strongly agree":

Q6 "I would have the right words to say if a patient mistreated a member of the medical team."

Strongly Disagree 1 2 3 4 5 Strongly agree

Q7 "I would have a plan for what to do if a patient mistreated a member of the medical team."

Strongly Disagree 1 2 3 4 5 Strongly agree

Q8 "If I wanted to bring up an instance of patient mistreatment with my supervisor, I would feel comfortable doing so."

Strongly Disagree 1 2 3 4 5 Strongly agree

Q9 "There is something I can do to address mistreatment of medical professionals by patients."

Strongly Disagree 1 2 3 4 5 Strongly agree

Q10 "Patient mistreatment of the medical team by patients has an impact on the quality of patient care."

Strongly Disagree 1 2 3 4 5 Strongly agree

Q11 "I am aware of an institutional procedure for addressing mistreatment of medical professionals by patients."

Strongly Disagree 1 2 3 4 5 Strongly agree

Q12 "I would be willing to speak up for myself if I was being mistreated by a patient."

Strongly Disagree 1 2 3 4 5 Strongly agree

Q13 “I would be willing to speak up for others if I witnessed them being mistreated by a patient.”

Strongly Disagree 1 2 3 4 5 Strongly agree

Q14 “I would feel comfortable de-escalating a hostile patient.”

Strongly Disagree 1 2 3 4 5 Strongly agree

Q15 “I am aware of evidence-based strategies for de-escalating a hostile patient.”

Strongly Disagree 1 2 3 4 5 Strongly agree

<u>Multiple Choice and Free Response Question:</u>

Please choose between A, B, and C to finish the following statement, and explain your reasoning in the free response area.

Q16 If a patient started mistreating me in the presence of my supervisor, I would prefer...

A. That my supervisor address the behavior immediately.

B. That my supervisor only address the behavior if it appeared I would benefit from their support.

C. Something else.

Q17 Please explain you reasoning below:
